# Medical students’ perception of supervision in MedUniVienna’s structured internal medicine and surgery clerkship program: Subject-specific differences and clerkship sequence effects

**DOI:** 10.3205/zma001729

**Published:** 2025-02-17

**Authors:** Angelika Hofhansl, Gerhard Zlabinger, Lena Bach, Josefine Röhrs, Anna-Maria Mayer, Anita Rieder, Michaela Wagner-Menghin

**Affiliations:** 1 Medical University of Vienna, Teaching Center, Vienna, Austria; 2 Medical University of Vienna, Center for Public Health, Vienna, Austria; 3 Catholic University of Eichstätt-Ingolstadt, Faculty of Empirical Educational Sciences, Eichstätt, Germany; 4 Medical University of Vienna, Vice-rectorate for Education, Vienna, Austria; 5 Medical University of Vienna, Department of Psychiatry and Psychotherapy, Clinical Division for Social Psychiatry, Vienna, Austria; 6 Medical University of Vienna, Comprehensive Center for Clinical Neurosciences and Mental Health, Vienna, Austria

**Keywords:** clinical supervision, general clinical experience, medical education, undergraduate students, students’ perspective, workplace-based learning

## Abstract

**Background:**

Clerkships for supervised learning of clinical skills are part of modern medical curricula. The availability of clerkship placements in clinics and the provision of competent supervision are essential for effective work-based learning. The scheduling of compulsory and elective work-based learning opportunities for undergraduate medical students (UGMS), especially when their numbers are high, results in varying clerkship sequences, which can influence career plans and examination outcomes. The effect of different clerkship sequences on students' impressions of clinical supervision remains unclear. Therefore, this study describes subject-specific differences in students' perceptions of clinical supervision during surgical (SC) and internal medicine (IMC) clerkships and addresses the impact of varying clerkship sequences and increasing clinical experience thereon.

**Method:**

In this survey, 1,017 final-year students at the Medical University of Vienna (from 2015 to 2019) retrospectively evaluated the quality of supervision they received during the SC and IMC using a newly piloted questionnaire on supervisory roles.

**Results:**

Students described their supervisors as less likely to exercise the roles of gatekeeper/safeguarding, training, and mentoring during the SC than during the IMC. During IMC, the supervisory activities received most often were to ensure patient and trainee safety, whereas during SC, it was to ensure trainee safety and to teach techniques and procedures. Ensuring an appropriate level of clinical duty was the third highest priority in both clerkships. Students’ general clinical experience influenced how they perceived the supervision, with students completing SC later in their pathway reporting having received similar levels of supervision in both clerkships.

**Conclusions:**

Supervision experiences during the first clerkship appear to shape students’ expectations of subsequent supervision. Providing additional support to foster a strong supervisory relationship, tailored to meet the specific supervision needs of UGMS newly entering year 6, could benefit both supervisors and students.

## Zusammenfassung

**Hintergrund:**

Klinisch-praktische Tätigkeiten zum supervidierten Erlernen klinischer Fertigkeiten sind feste Bestandteile moderner medizinischer Lehrpläne. Die Verfügbarkeit von Praktikumsplätzen in Kliniken und die Bereitstellung einer kompetenten klinischen Supervision sind dabei für ein effektives Lernen am Arbeitsplatz von wesentlicher Bedeutung. Das Planen von verpflichtenden und frei wählbaren Praktika durch Medizinstudierende im Grundstudium (MSGS) führt insbesondere bei hohen Teilnehmer*innenzahlen zu unterschiedlichen Praktikumsabfolgen, die die Karriereplanung und den Studienabschluss beeinflussen können. Wie sich die unterschiedlichen Praktikumsabfolgen auf die Wahrnehmung der klinischen Supervision durch die Studierenden auswirken, ist unklar. Daher beschreibt diese Studie fachspezifische Unterschiede in der Einschätzung der klinischen Supervision durch die Studierenden während der Tertiale in Chirurgie (TC) und Innerer Medizin (TIM) und befasst sich mit den Auswirkungen unterschiedlicher Tertialabfolgen und zunehmender klinischer Erfahrung.

**Methode:**

In dieser Befragung evaluierten 1.017 Studierende im letzten Studienjahr der Medizinischen Universität Wien (von 2015 bis 2019) anhand eines neu erprobten Fragebogens zum Rollenverhalten der betreuenden Ärztinnen und Ärzte retrospektiv die Qualität der Supervision, die sie während des TC und des TIM im Rahmen ihres Klinisch-Praktischen Jahres im 6. Ausbildungsjahr erhalten haben.

**Ergebnisse:**

Die Studierenden gaben an, dass ihre Betreuer:innen während des TC die Rollen Gatekeeping, Training bzw. Mentoring weniger oft einnahmen, als während des TIM. Während des TIM bestanden die Supervisionstätigkeiten am häufigsten darin, die Sicherheit der Patientinnen und Patienten und der Auszubildenden zu gewährleisten, während im TC die Sicherheit der Auszubildenden sowie die Vermittlung von Techniken und Verfahren im Vordergrund standen. Die Sicherstellung eines angemessenen Niveaus der klinischen Aufgaben für die Studierenden hatte in beiden Praktika die dritthöchste Priorität. Die klinische Vorerfahrung der Studierenden hatte Einfluss darauf, wie sie die Supervision wahrnahmen, wobei Studierende, die das TC später im Klinisch-Praktischen Jahr absolvierten, berichteten, dass ihnen in beiden Praktika ein ähnliches Maß an Supervision zuteil wurde.

**Schlussfolgerungen:**

Supervisionserfahrungen während der ersten Phase des Klinisch-Praktischen Jahres scheinen die Erwartungen der Studierenden an die anschließende klinische Supervision zu prägen. Die Bereitstellung zusätzlicher Unterstützung zur Förderung einer guten Supervisionsbeziehung, die auf die speziellen Supervisionsbedürfnisse von MSGS zugeschnitten ist, die neu in das 6. Jahr eintreten, könnte sowohl den Supervisor:innen als auch den Studierenden zugutekommen.

## 1. Introduction

Modern medical undergraduate curricula incorporate clerkships in which undergraduate medical students (UGMS) spend most of their time learning in the clinical workplace [1]. From a cognitivist perspective, the workplace is considered an ideal environment for learning complex skills because it provides opportunities to engage in authentic, real-life tasks of varying complexity [2]. However, as performing complex tasks alone without reflection and guidance is insufficient for acquiring medical skills [3], the social constructivist perspective [4] emphasizes the supervisory relationship and organizational aspects of workplace learning as influential for learning. 

Competent supervision is necessary for effective workplace learning [5], [6]. Supervision can be broadly defined as: a “[…] joint endeavor to improve the quality of trainees’ professional competence, their professional relationships (e.g., to clients, and co-workers), their personal development and ultimately to advance the wider profession” ([7], p.5). A more experienced member of a profession supervises younger colleagues [8]. A clinical supervisor plays three distinct roles: gatekeeping, training, and mentoring. The gatekeeping role involves overseeing the quality of the professional services that trainees provide to patients. The training role emphasizes developing trainee skills and competencies, while the mentoring role focuses on supporting their integration into the workplace [8], [9], [10], [11]. In a successful supervisory relationship, UGMS feel safe to focus on learning while engaging in work [12], [13], agree with their supervisors on whether direct or indirect supervision is required, and when feedback should be given or sought [13].

To make such a supervisory relationship effective, a structured educational framework should be established, including logbooks and portfolios with clearly defined learning goals, and the requirement to jointly document the learning process [6], [14]. Additionally, adequate value should be placed on the role of medical students by promoting communities of practice. Notably, given the importance of the proper scheduling of individual study modules in the curriculum, aligning workplace-based learning opportunities can present marked challenges, particularly when accommodating numerous UGMS. The inevitably resulting variations in clerkship subject sequences can influence career plans [15], [16] and affect students’ National Board of Medical Examiners' subject examination scores [17]. Students with prior clerkship experience tend to perform better in internal medicine [17] and surgery [18] examinations. Furthermore, Cottrel et al.’s [5] qualitative study on the experiences of postgraduate medical trainees and their supervisors revealed that “supervisory practice was very variable” ([5], p.1047) between specialties. In fields such as surgery and anesthetics, providing necessary supervision during trainee-performed procedures appears to be widespread. Conversely, in areas such as general medicine, internal medicine, pediatrics, and psychiatry, supervision occurs predominantly during scheduled educational sessions. Notably, this assertion is based on a small sample size (n=2), as the study was qualitative in nature. These findings align with the learning objectives [19] for UGMS surgery and internal medicine clerkships (SC and IMC). SCs entail technical procedures that challenge students’ bi-manual and visuospatial skills, necessitating a “hands-on” approach with appropriate supervision during early-stage training. IMCs prioritize analytical and reasoning skills, requiring a discussion-oriented approach fulfilled via scheduled meetings. 

We use a framework comprising the three supervisory roles [8], [9], [10], [11] in workplace learning [4] as a lens to explore how UGMS perceive supervision in clerkships in different subjects. The framework provides guidance for exploring student perceptions of supervision within a structured educational program aimed at facilitating supervisors’ and students’ engagement in their professional roles [9], [14]].

This study addresses how UGMS perceive the supervision they receive, with a particular emphasis on subject-specific differences. Specifically, it explores the extent to which students perceive supervisors’ engagement in supervisory tasks associated with three roles – gatekeeper, trainer, and mentor – during IMCs and SCs. Additionally, this study examines how variations in general clinical experience resulting from different clerkship sequences affect UGMS perceptions of clinical supervision during SCs compared to IMCs.

## 2. Methods

### 2.1. Participants and setting

Potential participants were UGMS (2015–2017, *n*=1,712; *n_male_*=869; *n_female_*=753; *n_not specified_*=90), taking year 6 of the integrated organ-based undergraduate medical curriculum offered at Medical University Vienna, Austria. Year 6 includes three 16-week advanced clerkships (SC, IMC, and elective (X)). Students who completed SC and IMC in Austrian hospitals (n=1,017) were included, out of which n=1005 completed ratings for both clerkships. Over 100 contracted teaching hospitals provide clerkship positions, with 1:1 supervision given by residents or senior doctors. Supervisors undergo four-hour training covering legal and organizational duties, the importance of each supervisory role, and how to work with the standardized student logbook and portfolio. Students organize their clerkship trajectory based on preferences and availability; as such, six different patterns are observable. The pattern starting with SC followed by X and then IMC was the least preferred pattern (11% of students), while the pattern starting with IMC followed by SC then X was the most preferred (23%), as shown by the significant OneSampleChiSquare-test (χ*^2^*(5, N=1005)=64.033, *p*<.001).

### 2.2. Measures

#### 2.2.1. Perceived supervisory engagement

To assess to what extent UGMS perceive their supervisors taking the role of gatekeeper, trainer, and mentor, a self-administered supervisors’ role questionnaire was composed by translating supervisory activities as listed by Grant et al. [20] and revising them to fit the context or to reduce content overlap (done by AH, GZ, MWM). Students rated the extent to which they received each supervisory activity using a four-point rating scale ((1) *“not at all”*, (2)* “to a small extent”,* (3) *“to a relevant extent”*, and (4) *“to a full extent”*) (see attachment 1 , questionnaire). A four-factor structure, with gatekeeping/safety (3 items), gatekeeping/assessment (5 items), training (5 items), and mentoring (7 items) based on the supervisory roles’ framework [8], [9], [10], [11] could be replicated by exploratory and confirmatory factor analyses in two independent samples. The questionnaire, its instructions, and details on its development and psychometric evaluation can be found in the attachment 1 . Scale reliability was between acceptable and good (Cronbach’s α=0.65-0.92).

#### 2.2.2. Clinical experience

To take the increasing clinical experience into account, students indicated when, in their trajectory, they completed the rated clerkship (first, second, and third clerkship periods). With three clerkship periods, six sequence patterns were possible. Consequently, surgical experiences gained in a period prior to the IMC may influence perceptions of supervision in internal medicine and vice versa. 

#### 2.2.3. Control variables

Cohort-specific effects were controlled for by coding the year in which participants took the end-of-year exam. Hospital effects were controlled for by coding hospital size using official information on bed numbers, and categorizing as follows to build groups of similar size: 1=up to 350 beds, 2=351 to 650 beds, 3=651 to 1000 beds, 4=more than 1000 beds. Smaller hospitals are often contracted as teaching institutions, while larger hospitals are typically affiliated with universities.

### 2.3. Procedure

Data were collected during the evaluation week that followed the completion of all students’ clerkships. Students completed the questionnaire for both clerkships (IMC and SC). Prior to receiving the materials, the students were notified of their participation in an anonymous survey. Returning completed materials for a minimum of one clerkship was considered consent to participate. 

### 2.4. Statistical analysis

#### 2.4.1. Supervisor engagement in supervisory activities as perceived by the UGMS 

We assessed UGMS perceptions of their supervisors’ engagement in supervisory roles in the SC and IMC using the mean scale score of the supervisors’ role scales. We also examined the relative frequency of receiving supervision (not received/received) and the cumulative frequencies of the extent (small, relevant, full) of receiving supervision for each activity, providing item-wise means and standard deviations for IMC and SC ratings. We compared means using the paired Wilcoxon signed-rank test for item-wise comparisons and the paired t-test for scale scores. Additionally, we transformed item-wise means into z-scores and created a bar chart to identify the most-received activities in each clerkship.

#### 2.4.2. Influence of clinical experience on the perception of supervision during clerkship 

We conducted a mixed analysis of variance (mixANOVA) to assess how clinical experience affected UGMS perception of clinical supervision during clerkship. The between-subject factor “clerkship sequence” was constituted by the six different possible sequences for completing the two mandatory subjects and the elective subject. The within-subject factor was “clerkship theme”, comprising internal medicine and surgery. Scale scores, representing the supervisory roles (gatekeeping/safety & assessing, training, mentoring) established during the pilot questionnaire’s psychometric evaluation, were used as measures for the mixANOVA. Data analyses were performed using the Statistical Package for the Social Sciences (SPSS; Version 28, IBM). The Medical University of Vienna’s Board for Privacy Protection reviewed the study protocol and granted permission to process the data.

## 3. Results

### 3.1. Supervisor engagement in supervisory activities as perceived by the UGMS

The statistics summarizing UGMS perceptions of supervisor engagement revealed significant mean differences for the gatekeeping/safety, training, and mentoring scales (see table 1 (Tab. 1)). The students reported receiving those activities significantly less often during SC than during IMC, though the effects were small. The relative frequencies of receiving (not received/received) the single activities illustrated that safety activities were all received by more than 90% of students during both clerkships. The proportion of students receiving these activities fully was smaller. Approximately 84% to 91% of the students received the assessment activities, but fewer than 40% reported receiving them in full. During the IMC, all training activities were received by at least 90% of students, except for the activity “provide formal feedback”; three were received fully by about 40% of students. During the SC, two activities were received by at least 90% of students, and only ‘teaching specific techniques and procedures’ was received fully by 48% of students. The seven mentoring activities were all received by a proportion of students exceeding 80%; only the mentoring activity “share professional experience” was received fully by over 40% of students (see table 1 (Tab. 1)).

**Figure 1:**
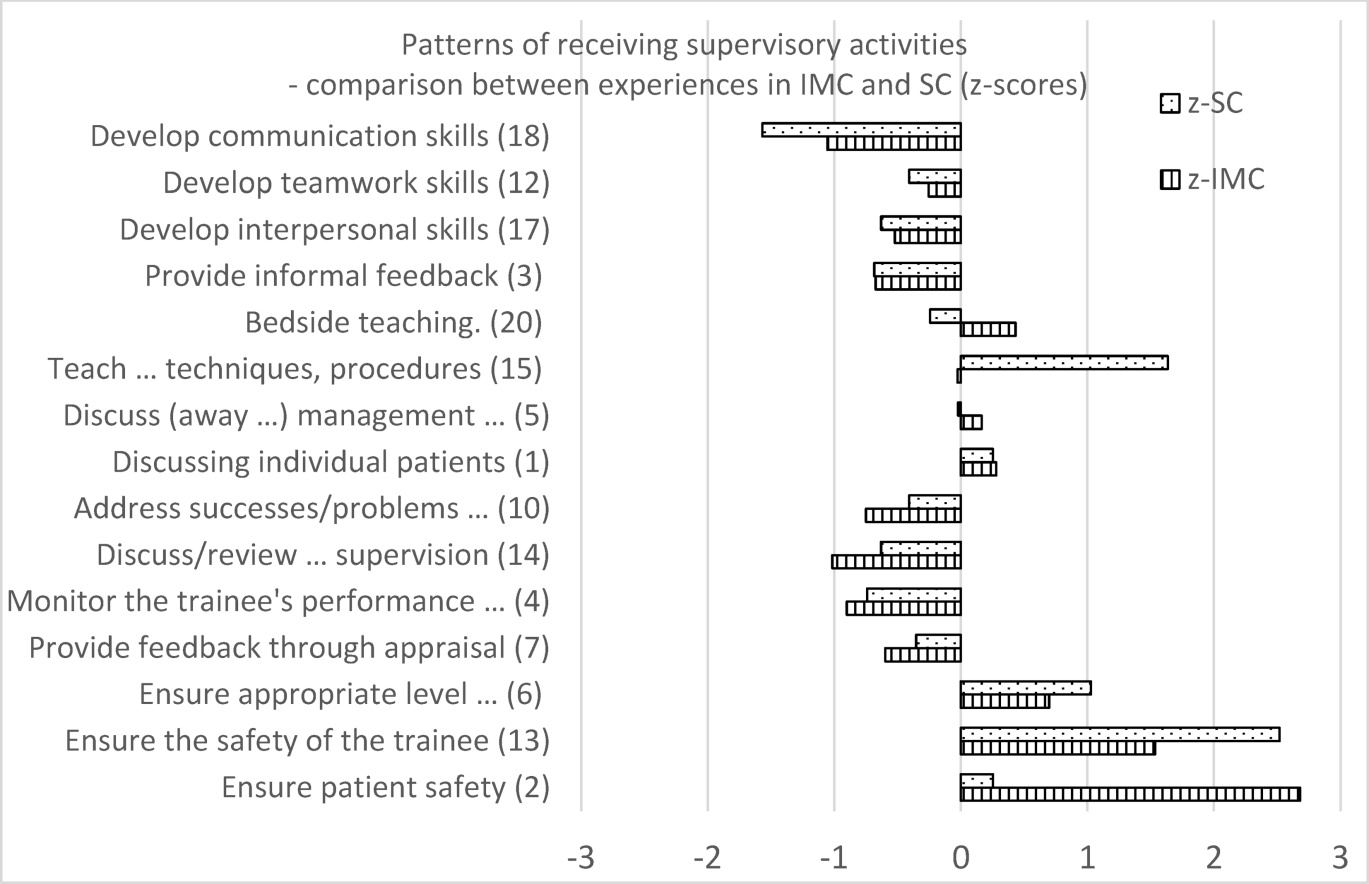
Patterns of receiving supervisory activities z-SC: z-values surgery clerkship; z-IMC: z-values internal medicine clerkship

A comparison of undergraduates’ perceptions of supervisory activities between clerkships showed that the pattern of received activities differed between the two clerkships (see figure 1 (Fig. 1)). During the IMC, the highest priority was to ensure patient safety (*z*=2.68), and the second highest was to ensure safety of the trainee (*z*=1.54). During the SC, the highest priority was to ensure trainee safety (*z*=2.52), and the second highest priority was to teach techniques and procedures (*z*=1.64). Ensuring an appropriate level of clinical duty was the third highest priority in both clerkships.

Students thus perceive their IMC and SC supervisors not to engage to a “full” extent in their roles and to enact their roles differently in each clerkship. While IMC supervisors prioritize “ensuring patient safety” and “ensuring trainee safety”, SC supervisors prioritize “trainee safety” and “teaching-specific techniques and procedures”.

### 3.2. Influence of clinical experience on students’ perceptions of clinical supervision 

The mixed ANOVA revealed a significant interaction effect of “clerkship sequence” and “clerkship theme” on the perception of clinical supervision, as measured by the four supervisory role scales (*F_Wilks-Lambda_*(5, 999)=2.482, *p*<.001, *η_p_*^2^=.012) (see table 2 (Tab. 2), row SxT). This effect indicates that students’ clinical experiences influenced how they perceived the provision of supervisory activities in the two thematic clerkships. Including the date of participating in the evaluation week (year) as a covariate in the model did not change this result. Thus, we continued the analysis without this covariate. 

The students’ supervisory experiences in their IMCs were compared with those in SCs using repeated measurement tests for each clerkship trajectory to further explore the interaction effect (see table 2 (Tab. 2), simple main effects – between): Significant results for all trajectories except IMC_X_SC were found. This indicates that only the students who took their SC after their IMC and elective clerkships perceived the provision of supervision to be equivalent in both compulsory clerkships. According to the descriptive results (see table 3 (Tab. 3)), this seems to be similar for SC_IMC_X students. Descriptive results further indicate that students in the X_SC_IMC trajectory experience more safety, training, and mentoring activities in their last clerkship, whereas IMC_SC_X students seem to experience less of all of the supervisory activities in their second clerkship (see table 3 (Tab. 3)).

To summarize, we found evidence that prior clinical experience, as established through different clerkship sequences together with subject effects, shapes how students perceive the supervision received in later following clerkships.

## 4. Discussion

Effective supervision is crucial for workplace-based learning, particularly for UGMS, as they develop essential clinical skills during clerkships, and the quality of clinical supervision markedly influences their study behavior [21]. Using a framework comprising the three supervisory roles [8], [9], [10], [11] in workplace learning [4], we described how UGMS, studying in a curriculum designed to provide an ideal workplace-based learning environment supported by a clinical logbook and portfolio, perceived clinical supervision relative to gatekeeping, training, and mentoring roles. 

UGMS reported experiencing most activities not to a “full” extent in both clerkships, thereby replicating the results published for postgraduate medical students (PGMS) [20]. The two activities that were received to a “full” extent by most students (>60%) were the gatekeeping/safety activities focused on patient and student safety. Despite the clear structure and instructions outlined in the logbook, only a small percentage of students felt that they had fully received the supervisory activities related to the gatekeeping and feedback roles. Lacking more qualitative information, we can only speculate about the cause. Students may receive feedback during scheduled sessions but would have wished for more feedback, i.e., informally in everyday clinical practice. Teachers may have provided feedback but might not yet have been able to fulfill their role (i.e., were unable to verbalize their feedback in a helpful manner). Future mixed-method studies should address these gaps. 

IMC and SC supervisors were also found to prioritize supervisory activities differently, with the first prioritizing patient and trainee safety and the latter emphasizing trainee safety while teaching specific techniques and procedures. These subtle differences in prioritizing supervision are well reflected in the different learning objectives specified for each clerkship. 

Comparing undergraduates’ experiences of activities with PGMS’ experiences (specialist registrars), as published by Grant et al. [20], shows that the pattern of supervision received differed between the educational levels. In our study, UGMS perceived gatekeeping/safety to have a higher priority than PGMS did. Supervision priorities also differ in gatekeeping/assessment roles. Receiving feedback, being monitored, and discussing problems with performance are given a higher priority among PGMS compared to our results in UGMS in both clerkships. UGMS also received different training compared to PGMS. In our study, UGMS reported that discussing individual patients and off-bedside discussions about the management of specific illnesses had the highest priority among all the activities provided. PGMS reported receiving informal feedback with a higher priority [20] compared to UGMS in our study. 

The impact of increasing clinical experience, shaped by different clerkship subject patterns, on UGMS perceptions of clinical supervision was also explored. We found hints that prior clinical experience together with subject effects influence students’ perceptions of supervision received in later clerkships. Some students who completed the SC early in their program reported limited coverage of training activities compared to their IMC. Conversely, this was not the case for groups completing the SC late. Additionally, students completing the SC late perceived equivalent levels of mentoring in both clerkships. These results corroborate previous findings that medical students at lower educational levels prefer more direct supervision to avoid feeling overwhelmed by responsibility, unlike students at higher educational levels [22], [23]. Surgical departments accepting students to start their Year 6 should consider the specific supervision needs of beginners.

The clinical workplace is busy and has multiple responsibilities for supervisors. Even in university hospitals, supervisors struggle with balancing patients’ and students’ needs [9], [24], [25]. Students should be explicitly informed about their supervisors’ responsibilities and supervision priorities. Assigning clinical work appropriate to their level of training ensures they can perform safely while gradually being challenged to expand their abilities and fully integrate into the clinical routine. The university’s framework for clerkships currently schedules a compulsory onboarding meeting between supervisors and trainees to discuss the process of supervision. Enhancing such meetings with a standardized guide, similar to a patient information sheet, might be the easiest way to support both parties’ needs and prevent a gap between students’ and supervisors’ expectations.

### 4.1. Limitations

Verbally anchored rating scales capture respondents’ subjective experience in situations. In our case, this is the extent of experienced supervisory activities, as defined by three supervisory roles. Our results do not provide objective measures of the frequency or quality of supervisors’ engagement. However, following constructivist-based supervisory role theory [8], [9], [10], [11], [26], this prioritization of subjective experience above objective observation is not a limitation, but the very phenomenon one should seek to study. In human interactions, such as a supervisory relationship, expectations related to enacting a role may vary between students and supervisors and need to be discussed explicitly to facilitate mutual understanding. Future studies interested in objective measures of frequency could use direct observation of activities or a study with an explicit frequency rating.

We could not obtain data on supervisors’ experience or supervisory conditions in hospitals or data on students’ year-5 exam scores due to concerns about data privacy. Additionally, data collection occurred only after all clerkships were completed and without controlling for sequence effects in questionnaire completion. On the positive side, having students complete the ratings for both clerkships at a single time point may have prevented response shift bias, which can occur when new experiences between two measurement points alter a participant’s frame of reference. Future studies should explore methods for gathering anonymized information on supervisory conditions and exam scores, and collect data on supervisory experience upon completion of each clerkship period. 

As this is the first study to use a newly piloted questionnaire to explore subject-specific perceived supervision differences in a multi-site undergraduate structured clerkship program, we limited our exploration to subject-specific differences within the same program. UGMS in clerkship programs with different structures might have different perceptions, which could be explored in the following studies. Further disentangling the effects of increasing experience, clerkship subject, and subject preference requires an experimental study with a random allocation of clerkship trajectories.

## 5. Conclusion

Students’ clinical experiences impact how they evaluate the supervision received during each clerkship. Because supervisory practices vary markedly and UGMS receive subtly different supervision compared to PGMS, as well as in IMCs and SCs, clinical supervisors should be equipped with strategies to identify UGMS’ specific needs [27].

Developing a supervisor guide for optimal UGMS supervision has already been suggested [28]. This guide must provide specific details on how to best tailor the provision of supervision to students’ needs within each clinical specialty and illustrate successful models for managing the relationship with the student in each supervisory role [29], [30]. The corresponding UGMS guide for effective clinical workplace learning must highlight potential training opportunities and outline the expected supervision, considering the demands of the clinical environment.

## Notes

### Authors’ contributions

AH, GZ, AR, and MWM worked on the conception and design of the work and the current manuscript. Data acquisition and management were performed by AH, GZ, LB, JR, and AMM. The analysis was performed by MWM and AMM. For data interpretation, as well as for drafting and revising the manuscript, the team of AH, GZ, LB, AMM, and MWM worked closely together. All authors approved the submitted version and agreed to be personally accountable for the author's own contributions.

### Authors’ ORCIDs

- Angelika Hofhansl: [0000-0003-1248-7840]
- Gerhard Zlabinger: [0000-0002-7478-4173]
- Anna-Maria Mayer: [0000-0002-8562-5418]
- Michaela Wagner-Menghin: [0000-0003-1645-7577]

### Ethics approval and consent to participate

The Medical University of Vienna’s Board for Privacy Protection reviewed the study protocol and granted permission to conduct the study. All participants provided informed consent to participate. All methods were carried out following relevant guidelines and regulations.

### Availability of data and materials

The data that support the findings of this study are available from the corresponding author upon reasonable request.

## Acknowledgements

Pursuing this project was facilitated by many people. The authors owe special thanks to several data typists working on entering the complex data. We would also like to express our thanks to all the students who provided their experiences.

## Competing interests

The authors declare that they have no competing interests. 

## Supplementary Material

Questionnaire, instructions and details

## Figures and Tables

**Table 1:**
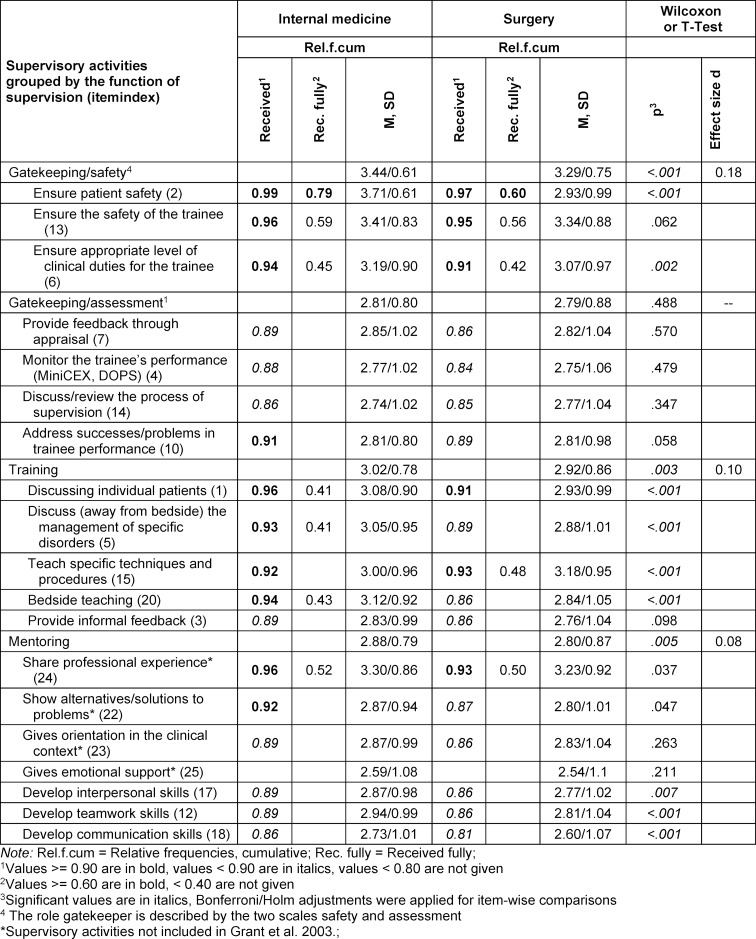
Supervisors’ engagement in supervisory roles Means, SDs for scales and items; (cumulative) relative frequencies for supervisory activities, N=1017

**Table 2:**
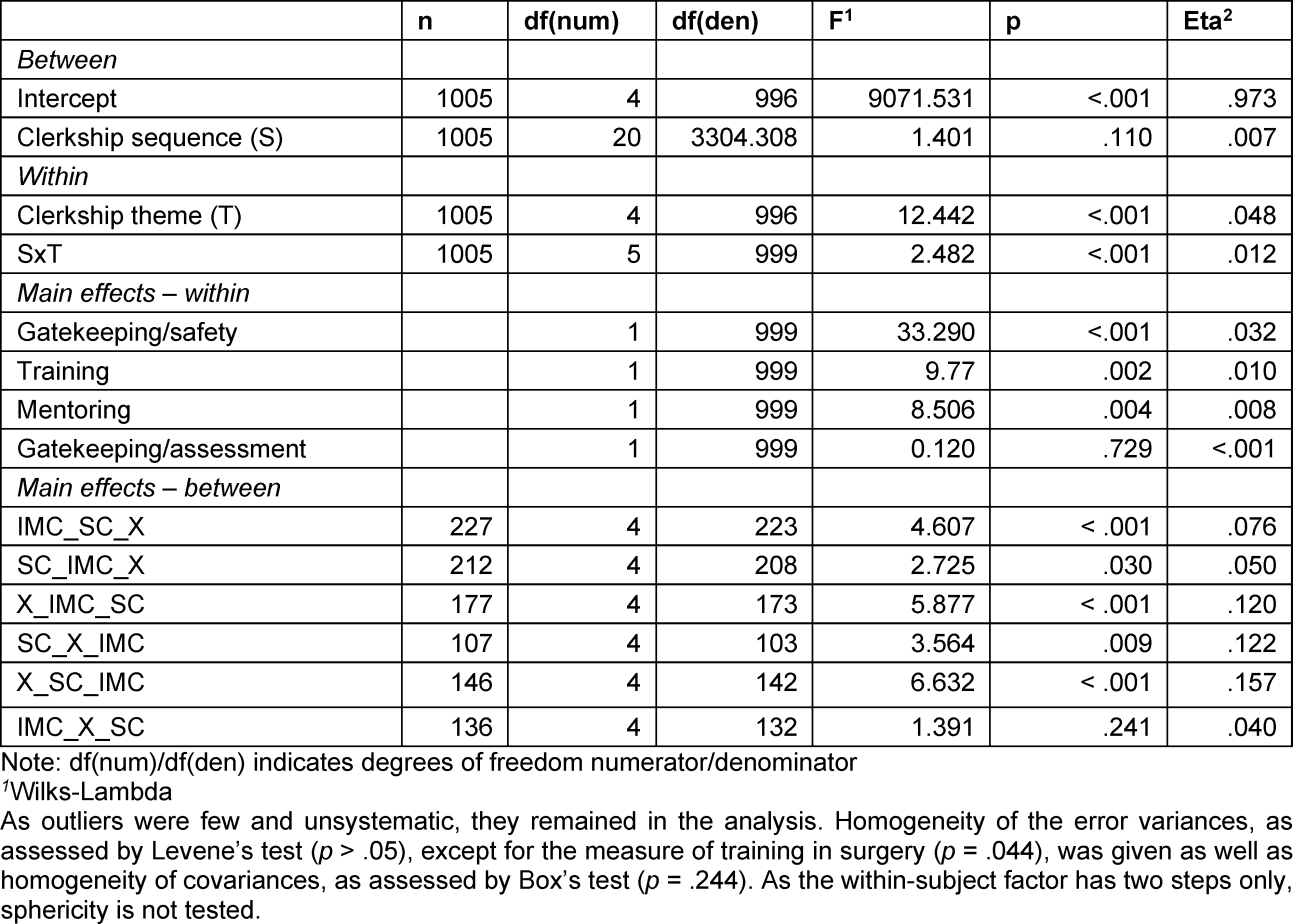
Mixed ANOVA results – supervisory functions scales

**Table 3:**
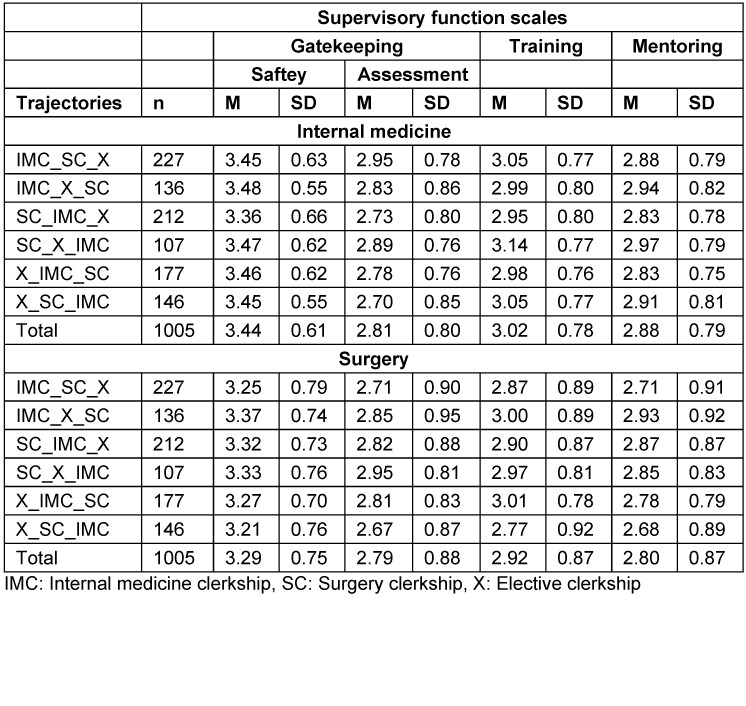
Clerkship sequences: Means and standard deviations of model variables and groups
