# Supplementary material for: Medical students’ perception of supervision in MedUniVienna’s structured internal medicine and surgery clerkship program: Subject-specific differences and clerkship sequence effects
Source: GMS J Med Educ. 2025 Feb 17;42(1):Doc5. doi: 10.3205/zma001729 (PMC12086254; doi:10.3205/zma001729)
Supplement: Questionnaire, instructions and details [file JME-42-5-s-001.pdf]

Attachment 1: Questionnaire, instructions and details

Questionaire English translation (German orginal version see Anhang 1)

To what **extent** has your clinical supervisor engaged in the following activities<br>☒ *Choose from the following answers* →<br>**He/She …**		**not at all**	**to a small extent**	**to a relevant extent**	**to a full extent**		
… discussed individual patients with me.		0	1	2	3	*1T*	Itemindex nur für Auswertung
… ensured patient safety. (No danger aroused for the patient due to negligence or intentional action).		0	1	2	3	*2S*	
… provided informal feedback on a regular basis.		0	1	2	3	*3T*	
…monitored my performance by means of compulsory evaluations (MiniCEX, DOPS).		0	1	2	3	*4A*	
… discussed (away from bedside) the management of specific disorders with me.		0	1	2	3	*5T*	
… ensured an appropriate scope and difficulty level of clinical duties for me.		0	1	2	3	*6S*	
… provided feedback through appraisal during scheduled meetings (midterm- and endmeeting, MiniCEX/DOPS-meetings).		0	1	2	3	*7A*	
… addressed successes/problems in my performance.		0	1	2	3	*10A*	
… developed my teamwork skills (my capability to integrate into a team).		0	1	2	3	*12M*	
… ensured my safety (in the workplace).		0	1	2	3	*13S*	
… discussed/reviewed the process of supervision with me.		0	1	2	3	*14A*	
… taught me specific techniques and procedures.		0	1	2	3	*15T*	
… developed my interpersonal skills (supported me in dealing with patients and colleagues).		0	1	2	3	*17M*	
… developed my communication skills.		0	1	2	3	*18M*	
…did bedside teaching.		0	1	2	3	*20T*	
… showed me alternatives/solutions to problems.		0	1	2	3	**22M*	
… gave me orientation in the clinical context.		0	1	2	3	**23M*	
… shared professional experience with me.		0	1	2	3	**24M*	
… gave me emotional support.		0	1	2	3	**25M*	
Which of these activities do you consider most helpful to your learning in the clinical clerkship in the subject ………………. ?	↑ *Please mark a maximum of 5 activities* ☒						
How satisfied were you in working with your supervisor for the clinical internship in the subject ....?							
**Very dissatisfied** -3 | -2 | -1 | 0 | 1 | 2 | 3 **Very satisfied**							

Attachment 1 to Hofhansl A, Zlabinger G, Bach L, Röhrs J, Mayer AM, Rieder A, Wagner-Menghin M. *Medical students' perception of supervision in MedUniVienna's structured internal medicine and surgery clerkship program: Subject-specific differences and clerkship sequence effects.* GMS J Med Educ. 2025;42(1):Doc5. DOI: 10.3205/zma001729

Development and psychometric evaluation of the perceived supervisory role-taking questionnaire

Twenty-one supervisory activities as listed by Grant et al [21] were translated to German. During revision by faculty (AH, GZ, MWM) six activities were dropped as irrelevant for the country's context or because of content overlap. After categorizing the activities in gatekeeping (n=7), training (n=5) and mentoring activities, four were added to better cover the mentoring role (n=7).

The ratings of SC and IMC experiences from 92 students (not included in the main study) were randomly assigned to form two parallel samples including only one rating per student and one observation per hospital, to preclude unwanted and uncontrollable correlation in the data. Exploratory factor analysis (EFA, principal component analysis with varimax rotation) was performed on one sample. The sample size of n=92 is suitable to conduct EFA extracting four factors out of 19 items under the assumption of high item communalities (between 0.60 and 0.80) [RA1]. Models including one to five factors were compared regarding their interpretability in light of the three supervisory roles. A four-factor model, comprising the factors gatekeeping/safety (three items), gatekeeping/assessing (four items), training (five items), and mentoring (seven items), explaining 68% of variance, proved interpretable, with high values of Cronbach's alpha between 0.65 and 0.91. A confirmatory factor analysis (CFA) was conducted using the other sample to further test the plausibility of the four-factor model. Findings indicating that the required sample size in CFA is dependent on aspects such as the number of factors, the number of indicators/factor and the magnitude of the factor loadings [RA2], found that for 3 (or more) factor models with 6 high loading indicators, samples of about n=100 are sufficient. Bollen-Stine Bootstrap p, conducted to account for violation of the multivariate normal distribution (as indicated by the Mardia test), indicated general model acceptance. The fit statistics root mean square error of approximation (RMSEA) and comparative fit index (CFI) yielded values, that in comparison with established thresholds, indicated the model to be non-fitting; but the standardized root mean residual (SRMR) indicated the model to be fitting. Running an EFA and CFA with crossed samples yielded similar results. We thus concluded that the newly composed supervisory role scales are valid and reliable for a detailed assessment of students' perception of supervision during clerkship (Results in table A1).

References for attachment

RA1 Mundfrom DJ, Shaw DG, Ke TL. Minimum Sample Size Recommendations for Conducting Factor Analyses. Int J Test. 2005;5(2):159-168. DOI: 10.1207/s15327574ijt0502_4

RA2 Wolf EJ, Harrington KM, Clark SL, Miller MW. Sample Size Requirements for Structural Equation Models: An Evaluation of Power, Bias, and Solution Propriety. Educ Psychol Meas. 2013;76(6):913-934. DII: 10.1177/0013164413495237

Table A1: Results of explorative factor analysis (parallel sample 1) and confirmatory factor analysis (parallel sample 2). Post hoc calculated cross-over results are printed in the shaded cells

Four factor solution	Parallel sample 1<br>n = 92	Parallel sample 2<br>n = 92
**Exploratory Factor Analysis**		
Kaiser-Mayer-Olkin coefficient	0.897	0.925
Bartlett-test	χ^2 = 1093.8; df = 171; n.s.	χ^2=1269; df=171; n.s.
Explained variance	68%	72%
**Cronbach Alpha for factors**		
Gatekeeping/safety (3 items)	0.655	0.805
Gatekeeping/assessing (4 items)	0.808	0.855
Training (5 items)	0.853	0.914
Mentoring (7 items)	0.910	0.923
**Confirmatory Factor Analysis**		
Mardia-Test	60.111; *c.r.* = 10.205;	67.920; *c.r.* = 11.531;
BollenStineBootstap p	*p* = 0.050;	*p* = 0.060;
RMSEA [CI]	0.104 [0.087; 0.122]	0.101 [0.083; 0.118]
CFI	0.857	0.888
SRMR	0.0763	0.0599

Note: n.s.=not significant, s.=significant; Mardia-Test: c.r.=critical ration, can be interpreted as z-values; when the crirtical ratio is greater than 1.96 in magnitude, the data may not be normally distributed. BollenStineBootstrap p (Testing the null hypothesis that the model is correct; p values>0.05 indicates mdel fit). As a commonly accepted rule of thumb (Hooper 2008), threshold values indication a good model fit are given in the literature as follows: CI=confidence interval; RMSEA (root mean square error of approximation) should be smaller than 0.07 for small samples; CFI (comparative fit index) should be around 0.95, close to 1; SRMR (standardized root mean residual) should be smaller than 0.8 (HU & Bentler).
